# Antibacterial and Antibiofilm Activity and Mode of Action of Magainin 2 against Drug-Resistant *Acinetobacter baumannii*

**DOI:** 10.3390/ijms19103041

**Published:** 2018-10-05

**Authors:** Min Kyung Kim, Na Hee Kang, Su Jin Ko, Jonggwan Park, Eunji Park, Dong Won Shin, Seo Hyun Kim, Seung A. Lee, Ji In Lee, Seung Hyun Lee, Eun Gi Ha, Seung Hun Jeon, Yoonkyung Park

**Affiliations:** 1 Department of Biomedical Science, Chosun University, Gwangju 61452, Korea; charm5964@naver.com (M.K.K.); govlgovl_2414@hanmail.net (N.H.K.); ksj920708@hanmail.net (S.J.K.); fhhhhgfh@naver.com (E.P.); 2 Department of Bioinformatics, Kongju National University, Kongju 38065, Korea; for_quality@naver.com; 3 Jangseong High School, Jeollanamdo 57216, Korea; dkatlf6017@naver.com (D.W.S.); twinpearl@naver.com (S.H.K.); iceone256325@naver.com (S.A.L.); jiin1839@gmail.com (J.I.L.); dltmdzus4001@naver.com (S.H.L.); dwar77@naver.com (E.G.H.); bioman255@naver.com (S.H.J.); 4 Research Center for Proteineous Materials, Chosun University, Gwangju 61452, Korea

**Keywords:** *Acinetobacter baumannii*, multidrug-resistant, antimicrobial peptide, antibiofilm activity, physiological salt

## Abstract

Antimicrobial peptides (AMPs) are promising therapeutic agents for treating antibiotic-resistant bacterial infections. Previous studies showed that magainin 2 (isolated from African clawed fogs *Xenopus laevis*) has antimicrobial activity against gram-positive and gram-negative bacteria. The present study was conducted to investigate the antibacterial activity of magainin 2 against *Acinetobacter baumannii*. Magainin 2 showed excellent antibacterial activity against *A. baumannii* strains and high stability at physiological salt concentrations. This peptide was not cytotoxic towards HaCaT cells and showed no hemolytic activity. Biofilm inhibition and elimination were significantly induced in all *A. baumannii* strains exposed to magainin 2. We confirmed the mechanism of magainin 2 on the bacterial outer and inner membranes. Collectively, these results suggest that magainin 2 is an effective antimicrobial and antibiofilm agent against *A. baumannii* strains.

## 1. Introduction

The use of antibiotics for treating infections has resulted in the emergence of resistant strains [1]. New antibiotic-resistant strains are becoming a problem worldwide because conventional antibiotics cannot be administered. A recent report using data from the Infectious Diseases Society of America and hospital-based surveillance research refers to the pathogens as “ESKAPE” [2,3]. The ESKAPE pathogens include *Enterococcus faecium*, *Staphylococcus aureus*, *Klebsiella pneumoniae*, *Acinetobacter*, *Pseudomonas aeruginosa*, and *Enterobacter* species [4]. The ability of these bacteria groups to withstand antibiotic treatment is a major cause of hospital infections worldwide [5]. Notably, *Acinetobacter baumannii* has been reported as one of the most serious ESKAPE organisms resistant to antibiotics [6].

*Acinetobacter baumannii*, a gram-negative bacterium, is an opportunistic bacterial pathogen and has a high morbidity and mortality, especially in intensive care units with high pathogen infections. Infectious diseases include urinary tract infection [7], meningitis [8], skin infection [9], bacteremia [10], and pneumonia [11]. Many strains causing infection show resistance to antibiotics such as aminoglycosides, fluoroquinolones, colistin, β-lactams, and tetracyclines [12,13]. Also, *A. baumannii* is prone to biofilm formation and their ability to increase resistance to antibiotics.

Biofilm is a microbial aggregate formed by cells and cells that attach to each other and become embedded in a matrix of self-generated extracellular polymeric substances [14]. Biofilms can form on various abiotic surfaces including glass, polystyrene, and surgical instruments. They can also attach to tissues that are easily exposed to bacterial infections and cause problems [15]. Therefore, new drugs are needed to effectively treat antibiotic-resistant bacteria and biofilm-related infections. Antimicrobial peptides (AMPs) are excellent candidates for developing therapeutic agents.

AMPs are an essential component of innate immunity in host organisms, including animals, insects, plants, and humans [16]. Human host defense antimicrobial peptides are a major component of innate immunity and play an important role in preventing microbial infection and these peptides expressed in the skin, eyes, ears, and various tissues [17]. Among them, Defensins, LL-37 and Histatins are important to prevent oral cavity [18,19,20]. These molecules are amphipathic peptides typically composed of 12–50 amino acids and show antimicrobial activity against a broad spectrum of microorganisms including gram-positive and gram-negative bacteria, fungi, viruses, and cancer cells. These peptides contain arginine and lysine, which are positively charged and important in their mechanism of action [21]. In general, the bacterial membrane contains a higher content of negatively charged phospholipids compared to mammalian cells, enabling the positively charged peptide to bind more strongly with bacterial cells through electrostatic interactions. This destabilizes or disrupts the bacterial membrane and intracellular processes, leading to microorganism death.

Frog skin is the most abundant source of antimicrobial peptides [22] and secretes major peptides such as esculentin [23], temporin [24] and magainin [25] to protect against microbial invasion. AMPs and its analogue peptides detected in the skin of many frogs exhibit strong antimicrobial activity against antibiotic-resistant bacteria. We previously investigated magainin 2 (GIGKFLHSAKKFGKAFVGEIMNS), an antimicrobial peptide consisting of 23 amino acids isolated from the skin of the African clawed frog *Xenopus laevis* [25]. This peptide has been reported to exhibit broad antibacterial activity against gram-positive and gram-negative bacteria and anti-cancer activity against certain tumor cell lines [26,27].

In this study, we examined the antimicrobial activity of magainin 2 against *A. baumannii* strains and its toxicity towards mammalian cells. We also confirmed that the antibacterial activity of the peptide was maintained even under high-salt conditions. Next, we investigated the activity in a biofilm model, which is closely related to bacterial resistance. The mechanism of action of the peptide was confirmed by membrane-related experiments using *N*-phenyl-1-naphthylamine (NPN) and 3,3′-dipropylthiadicarbocyanine iodide (DiSC_3_-5). Our results suggest that magainin 2 can be used as an effective treatment for *A. baumannii* infections.

## 2. Results

### 2.1. Peptide Synthesis

**Figure 1:**
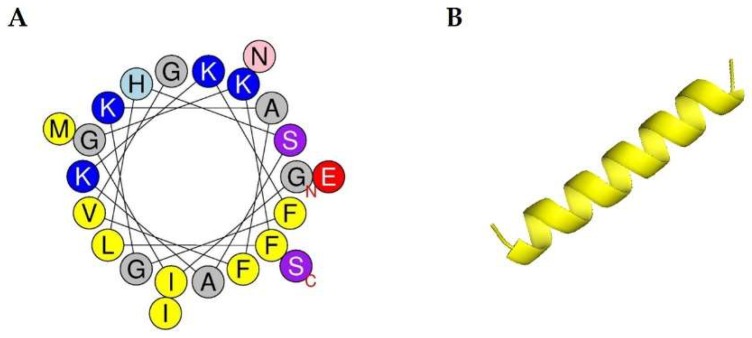
Structure analysis of magainin 2. (**A**) Helical wheel diagram of the peptide. The projection was obtained from http://heliquest.ipmc.cnrs.fr/cgibn/ComputParam.py. Positively charged residues are represented in blue, while hydrophobic residues are shown as yellow circles. The N-terminal and C-terminal parts are represented in red letters “N” and “C”. (**B**) Three-dimensional structure of magainin 2.

The magainin 2 sequence, observed molecular weight, hydrophobicity, hydrophobic moment, and net charge are summarized in Table 1. Magainin 2 is an antimicrobial peptide consisting of 23 amino acids with hydrophobic content, hydrophobic moment, and net charge of 0.373, 0.475, and 3, respectively. The wheel diagram and three-dimensional structure analysis predicted that the peptide contains hydrophobic residues (yellow circles) and forms an α-helix structure (Figure 1).

### 2.2. Circular Dichroism Measurements

**Figure 2:**
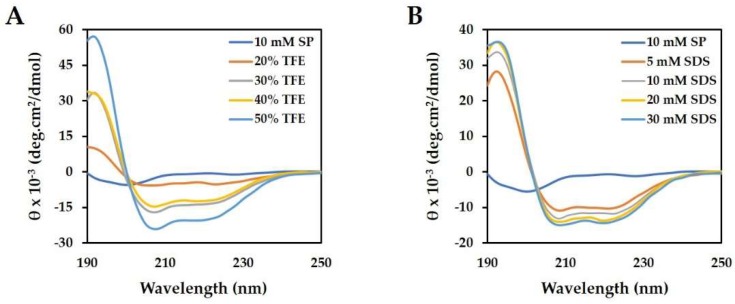
Circular dichroism (CD) spectra of magainin 2. (**A**) The peptide was measured in TFE, which mimics the hydrophobic environment of the microbial membrane and (**B**) SDS, which mimics the negatively charged prokaryotic membrane environment.

Based on the predicted results shown in Figure 1B; we measured the secondary structure of magainin 2 by circular dichroism (CD) spectroscopy. Structure analysis of magainin 2 was performed in various concentrations of trifluoroethanol (TFE) and sodium dodecyl sulfate (SDS) solutions (Figure 2). In 10 mM sodium phosphate buffer; the CD spectra of the peptide displayed a random coil. However; the peptide adopted a typical α-helical conformation with increasing TFE and SDS concentrations. The calculated α-helical contents of the peptide are shown in Table 2. As the TFE and SDS concentration increased; the α-helical contents increased from 6.4% to 50.8% and from 21.8% to 33.3%; respectively. These results demonstrate that magainin 2 forms a strong α-helix structure.

### 2.3. Antimicrobial Assay

The antimicrobial activity of the peptides against *A. baumannii* strains are summarized in Table 3. We compared the antibacterial effect of magainin 2 with those of melittin, buforin 2, ciprofloxacin, and gentamicin. Magainin 2 showed strong antibacterial activity with minimum inhibitory concentrations (MICs) of 4 and 2 µM against the standard strain (Korea Collection for Type Cultures (KCTC) 2508) and drug-resistant strains, respectively. This is similar to the MIC of melittin, which is known to have strong antibacterial activity. The activity of buforin 2 was 8-fold lower, ranging from 8 to 16 µM. Antibiotics showed activity against drug-resistant strains at 128 µM. Particularly, gentamicin showed low antibacterial activity with an MIC of 256 µM.

### 2.4. Cytotoxicity Assay

**Figure 3:**
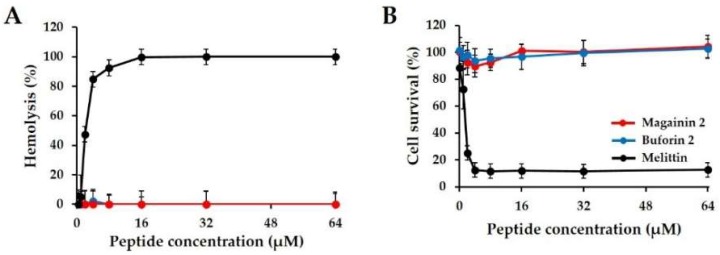
Cytotoxicity and hemolytic activity of magainin 2. (**A**) Hemolytic activity of peptides against mouse red blood cells (RBCs). (**B**) Cytotoxicity of peptides against HaCaT cells.

Hemolysis and cell viability assays were conducted to measure the toxicity of the peptides in mammalian cells. As shown in Figure 3A, melittin, a positive control peptide, induced more than 50% hemolysis at concentrations of 1–2 µM. In contrast, magainin 2-treated cells showed no hemolysis at 64 µM. We confirmed the cytotoxicity of magainin 2 in HaCaT cells. The curve in Figure 3B shows that high concentrations of magainin 2 exhibited low cytotoxicity of 0%, while melittin exhibited 100% toxicity at 2 µM. These results confirm that magainin 2 is not toxic to cells.

### 2.5. Biofilm Inhibition Assay

**Figure 4:**
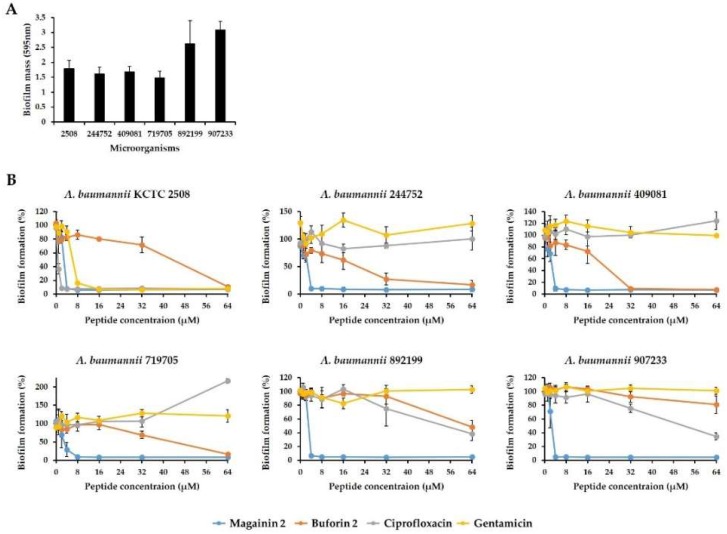
Effect of *Acinetobacter baumannii* biofilm formation by magainin 2. (**A**) Quantitative measurements of biofilm formation using crystal violet staining. (**B**) Inhibitory effect of peptides on biofilm formation. Antibiotics (ciprofloxacin and gentamicin) and buforin 2 were used as controls.

**Figure 5:**
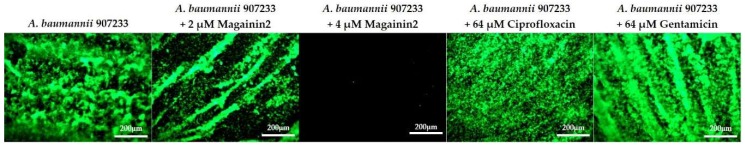
EVOS2 images of *Acinetobacter baumannii* biofilm stained with SYTO9 dye (green fluorescence).

To investigate whether magainin 2 can inhibit biofilm formation, the degree of biofilm formation by *A. baumannii* strains was confirmed in Mueller-Hinton broth (MHB) supplemented with glucose. As shown in Figure 4A, all strains formed biofilms and the *A. baumannii* 907233 strain had a biofilm mass of 3 at OD 595 nm. Compared to *A. baumannii* KCTC 2508 in the crystal violet staining assay, *A. baumannii* 907233 showed a 57.6% higher biofilm mass after 24 h of incubation (Figure 4A). The ability of magainin 2 and antibiotics (ciprofloxacin, gentamicin) to prevent biofilm formation was compared. Magainin 2 and antibiotics inhibited the biofilm formation of *A. baumannii* KCTC 2508 at concentrations of 2–8 µM, whereas buforin 2 showed inhibition at 64 µM. In resistant strains, magainin 2 inhibited biofilm formation at a low concentration of 4 µM, while the antibiotics did not exhibit biofilm formation inhibition activity until 32 µM (Figure 4B). The biofilm biomass was determined using the green dye SYTO9 and detected with the EVOS AUTO2 fluorescence microscope. *Acinetobacter baumannii* 907233 formed biofilm showing strong green fluorescence (Figure 5). Following treatment of the biofilm with magainin 2, SYTO9 fluorescence decreased in a concentration-dependent manner, while this was not observed following treatment with the other antibiotics. This data shows that magainin 2 has strong antibiofilm activity against drug-resistant *A. baumannii*.

### 2.6. Biofilm Reduction Assay

**Figure 6:**
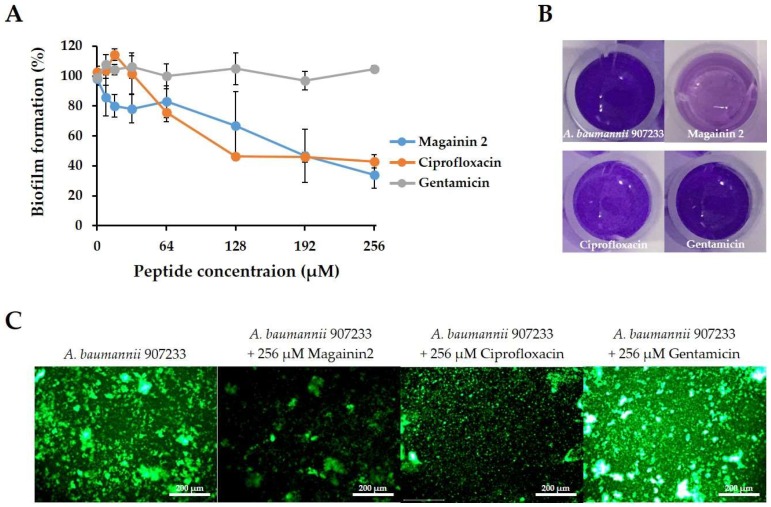
Biofilm reduction assay. (**A**) Degree of biofilm removal by magainin 2, ciprofloxacin, and gentamicin using crystal violet staining. (**B**) Image of removed biofilm after treatment with 256 µM of peptide and antibiotics. (**C**) SYTO9-stained biofilm image.

The biofilm reduction activity of magainin 2 was confirmed against *A. baumannii* 907233, which easily forms biofilm. Figure 6 shows the effect of the peptide and antibiotics on biofilm formed for 24 h. In the crystal violet staining assay, *A. baumannii* 907233 showed strong staining after incubation for 24 h (Figure 6B). Antibacterial treatment with magainin 2 and ciprofloxacin exhibited similar abilities to eradicate biofilm formation. The biofilm inhibition rates of magainin 2 were 33.3%, 53.4%, and 66.2% at 128, 192, and 256 µM, respectively. In contrast, gentamicin showed no biofilm inhibition effects at 256 µM (Figure 6A). Next, the biofilm was observed using SYTO9 (green dye). After 24 h, the biofilm stained with SYTO9 showed strong green fluorescence. The fluorescence was low in the presence of 256 µM magainin 2, indicating that it effectively disrupted the biofilm. Gentamicin showed no decrease in biofilm (Figure 6C). These results suggest that magainin 2 not only inhibits but also effectively eliminates biofilm.

### 2.7. Salt Sensitivity Assay

To investigate the effects of salts on antimicrobial activity, antimicrobial activity was measured at a physiological salt concentration (Table 4). As shown in Table 3, magainin 2 exhibited similar stability in the presence of physiological salts compared to buforin 2. The MIC values of *A. baumannii* KCTC 2508 and *A. baumannii* 907233 strain were 4 and 2 µM, respectively. In the presence of NaCl and FeCl_3_, magainin 2 activity was nearly maintained in both strains. In contrast, magainin 2 activity was slightly reduced to 8 µM in the presence of MgCl_2_.

### 2.8. Mechanism of Action Of Magainin 2

**Figure 7:**
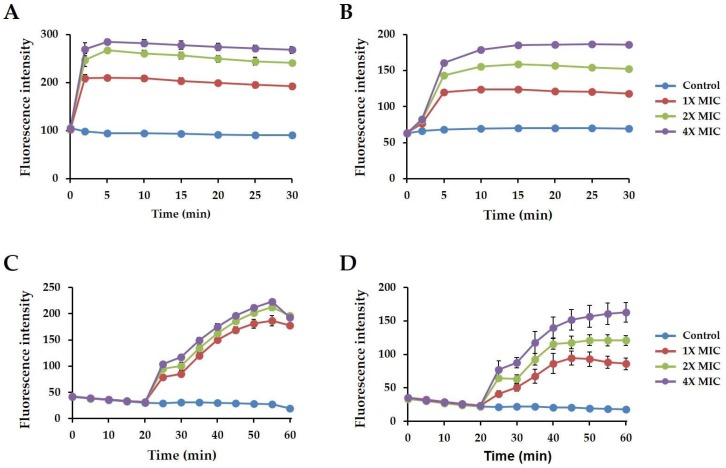
Magainin 2 mechanism of action. (**A**,**B**) The outer membrane permeability of magainin 2 was measured using NPN dye. (**C**,**D**) Depolarization of cytoplasmic membrane induced by magainin 2, determined using the membrane potential-sensitive fluorescent dye DisC_3_-5. (**A**,**C**: *Acinetobacter baumannii* KCTC 2508, **B**,**D**: *Acinetobacter baumannii* 907233).

To investigate the mechanism of magainin 2 activity towards drug-resistant bacteria, *A. baumannii* 907233 was used, while *A. baumannii* KCTC 2508 as a control strain. First, the outer membrane permeability of *A. baumannii* 907233 following treatment with magainin 2 was examined using NPN dye (hydrophobic fluorescent dye, exhibits fluorescence intensity in a hydrophobic environment). The bacterial outer membrane was disrupted, and the hydrophobic part of the membrane was exposed, after which NPN reacted to show fluorescence. As shown in Figure 7A,B, the intensity increased in a concentration-dependent manner, indicating that magainin 2 acts on the outer membrane. For *A. baumannii* KCTC 2508, the fluorescence intensity rapidly increased to 268, 246, and 209 within 2 min at 4×, 2×, and 1× MIC, respectively. In contrast, fluorescence increased to approximately 80 in *A. baumannii* 907233.

The ability to depolarize the *A. baumannii* 907233 inner membrane in the presence of magainin 2 was further confirmed using DiSC_3_-5. DiSC_3_-5 is a dye that accumulates in the cytoplasmic membrane of bacteria; the dye is released when the bacteria membrane is affected by the peptide. As shown in Figure 7C,D, addition of magainin 2 resulted in increased fluorescence in a dose-dependent manner, indicating the ability of magainin 2 to depolarize the membrane. The fluorescence intensity of *A. baumannii* KCTC 2508 was higher than that of *A. baumannii* 907233 strain and decreased at 60 min after peptide treatment. These results suggest that magainin 2 affects the inner membranes of both *A. baumannii* KCTC 2508 and 907233.

## 3. Discussion

Bacterial infections are among the major causes of death worldwide [28]. Conventional antibiotics were effectively used to treat pathogenic infections for half a century, but the emergence of antibiotic-resistant bacteria has made these infections difficult to treat [29,30]. Generally, long-term exposure to antibiotics leads to resistance. Antibiotics function against proteins that are essential to bacteria cells, and mutations in bacterial proteins cause antibiotic resistance. Bacteria can also transform antibiotics and make them inefficient by producing specific enzymes. Thus, an antibacterial with a new mechanism of action differing from existing antibiotics is urgently needed. Antimicrobial peptides have distinctive mechanisms and are emerging as new therapeutic agents.

AMPs are classified according to their α-helical, β-sheet, and random coil structures; the α-helical structure is the most common [31]. The predicted magainin 2 structure was confirmed by CD spectroscopy to be a-helical in the bacteria membrane, mimicking the surrounding environment, and appeared as a random coil structure in an aqueous environment. In increasing concentrations of SDS and TFE, the percent of a-helix content of magainin 2 also increased. This suggests that the α-helical structure enabled magainin 2 to interact with the bacterial membrane, which is related to its mechanism of action [32].

Because bacteria isolated from hospital patients are resistant to antibiotics, treatment of *A. baumannii* infection is becoming increasingly difficult [33]. Therefore, the activity of magainin 2 towards bacteria isolated from patients was evaluated. Magainin 2 exhibited lower antibacterial activity against drug-resistant *A. baumannii* than that observed against the susceptible *A. baumannii* (KCTC 2508) and exhibited more potent activity than antibiotics (ciprofloxacin and gentamicin). Thus, magainin 2 is a more effective therapeutic agent than antibiotics, which are not resistant to AMPs. Additionally, magainin 2 is not toxic to mammalian cells.

Biofilms are important for the survival of bacteria in the environment, and biofilm formation has been correlated with drug-resistance and several virulence factors [34,35]. The antibiotic resistance of biofilms is significantly higher than that of planktonic bacteria [36,37]. These biofilm-related infections are more difficult to control and can lead to death. *Acinetobacter baumannii* is a pathogen that commonly forms biofilm [38,39]. We analyzed the biofilm formation of *A. baumannii* strains treated with a peptide. Peptides and antibiotics showed similar abilities to inhibit biofilm formation by susceptible *A. baumannii*. However, in the five resistant strains, magainin 2 showed higher antibiofilm activity than antibiotics at low concentrations. These results suggest that magainin 2 exerts strong antibiofilm activity against drug-resistant *A. baumannii*. Furthermore, we evaluated the efficacy of magainin 2 on *A. baumannii* within established biofilms. Removing biofilms formed by pathogenic microorganisms is difficult. We found that magainin 2 was more effective than antibiotics towards removing formed biofilms. Thus, magainin 2 may be effective for treating biofilm-related infectious diseases.

The antimicrobial activity of most AMPs is strongly inhibited by presence of high ionic concentrations, which is a disadvantage as therapeutic agents in the serum or other body fluids [40]. For example, the activity of human β-defensins is influenced by NaCl in the airway surface liquid of cystic fibrosis patients [41]. In the presence of NaCl, MgCl_2_, and FeCl_3_ at concentrations similar to those in human bodily fluids, the activity of magainin 2 was maintained. These results demonstrate that magainin 2 is stable in high-salt environments, which did not affect the killing mechanism. Combined with previous toxicity data, this peptide may be useful for therapeutic applications in a physiological environment.

AMPs, which have cationic peptides, bind to the negatively charged bacterial membrane through electrostatic interactions [42]. Peptides can disrupt the membrane after binding or enter the membrane to form pores. Pore-forming mechanisms include models of “barrel-stave”, “carpet”, and “toroidal-pore” [42,43]. In a previous study, magainin 2 was shown to bind to the anionic lipid bilayers of the bacterial membrane via electrostatic attractions and form a pore, which eventually destroyed the membrane [44]. The peptide was suggested to form toroidal pores ~80 Å diameter in DMPC/DMPG liposomes [45]. Thus, we investigated the mechanism of magainin 2 against *A. baumannii* strains in this study.

To determine the mechanism of action against *A. baumannii* strains, we used NPN and DiSC_3_-5 dyes. First, damage to the bacterial outer membrane was monitored by measuring the fluorescence intensity of NPN. Magainin 2 induced an increase in fluorescence in both stains. Compared to the drug-resistant strain, the response of susceptible *A. baumannii* was faster. Depolarization of the bacterial cytoplasmic membrane was monitored by measuring the fluorescence intensity of DiSC_3_-5. Consistent with the results of the NPN test, the fluorescence intensity decreased at 55 min, indicating that magainin 2 rapidly affected the susceptible *A. baumannii*. Collectively, magainin 2 has an α-helical structure, which can associate with the *A. baumannii* membrane and uses a mechanism in which both the outer and inner membranes are affected.

## 4. Materials and Methods

### 4.1. Materials

SDS, trifluoroethanol (TFE), 3-(4,5-dimethylthiazol-2-yl)-2,5-diphenyltetrazolium bromide (MTT), dimethyl sulfoxide (DMSO), N-phenyl-1-naphthylamine (NPN), 3,3′-dipropylthiadicarbocyanine iodide (DiSC_3_-5), ciprofloxacin, and gentamicin were purchased from Sigma-Aldrich (St. Louis, MO, USA). Dulbecco’s Modified Eagle’s medium (DMEM), fetal bovine serum (FBS), and Dulbecco’s Phosphate Buffered Saline (DPBS) were obtained from Welgene (Daegu, Korea).

### 4.2. Microorganisms and Mouse Red Blood Cells

*Acinetobacter baumannii* KCTC 2508 was obtained from the KCTC. Other *Acinetobacter baumannii* 244752, 409081, 719705, 892199, and 907233 were antibiotic-resistant bacteria isolated from patients at Eulji University Hospital (Seoul, Korea). The study protocol was reviewed and approved by the institutional review board of Eulji Hospital (No. EMCS 2016). All patients gave their informed consent to participate in this study or the informed consent process was waived in accordance with the decision of the ethics committee of each hospital. The mouse used in this study was carried out in strict accordance with the recommendations in the Guide for the Care and Use of Laboratory Animals of the National Institutes of Health, and approved by the Committee on the Ethics of Animal Experiments (CIACUC2017-S0042; Chosun University, Gwangju, South Korea).

### 4.3. Peptide Synthesis and Sequence Analysis

The peptides were synthesized using the solid-phase-9-fluorenylmethoxycarbonyl method as reported previously [46] on a Rink amide 4-methylbenzhydrylamine resin using a Liberty microwave peptide synthesizer (CEM, Matthews, NY, USA). The purity and molecular weight of the peptide was confirmed by reversed-phase high-performance liquid chromatography and matrix-assisted laser desorption ionization-time of flight mass spectrometry. Projections of the predicted three-dimensional structures were constructed online using the Mobyle@RPBS bioinformatics portal (http://mobyle.rpbs.univ-paris-diderot.fr/cgi-bin/portal.py#welcome), whereas the HeliQuest site (http://heliquest.ipmc.cnrs.fr) was used to create helical wheel diagrams and determine the relative hydrophobic moments of the peptides.

### 4.4. Circular Dichroism Measurements

CD measurements were performed on a JASCO 810 spectropolarimeter (Jasco, Tokyo, Japan) using a 0.1-cm path length rectangular quartz cell [47]. The peptide structure was evaluated in various solutions. The solutions were prepared at a 40 µM peptide concentration in 10 mM sodium phosphate buffer, pH 7.2, to mimic an aqueous environment, TFE (20%, 30%, 40%, and 50% TFE) to mimic the hydrophobic environment of the microbial membrane, and SDS (5, 10, 20, and 30 mM SDS) as a negatively charged prokaryotic membrane-comparable environment. The spectra were recorded between 190 and 250 nm. The percent of α-helix content was calculated as follows:

% α-helix = −100 ([θ]_222_ + 3000)/33,000

### 4.5. Antimicrobial Activity Assay

The MIC of peptides was determined by the standard micro-dilution method [48] in 96-well microtiter plates. Briefly, *A. baumannii* strains were cultured in MHB media and prepared at 2 × 10^5^ colony-forming units per milliliter (CFU/mL). The peptides were serially diluted to concentrations between 1 and 32 µM in 10 mM sodium phosphate buffer in a 96-well plate. The bacteria were mixed with serially diluted peptide in the 96-well plate and incubated at 37°C for 18–24 h. The absorbance of the sample at 600 nm was measured using a microplate reader and repeated three times.

### 4.6. Hemolysis Assay

The hemolytic activity of peptides was evaluated using mouse red blood cells (RBCs). RBCs were washed three times with PBS (Phosphate Buffered Saline) until the supernatant was clear. The peptides were diluted to 64 µM and added to a 96-well plate. RBCs were added at a final concentration of 8% (*v*/*v*). After incubation for 1 h, the plate was centrifuged for 10 min and the absorbance of the supernatant was measured at 414 nm. The percentage of hemolysis was calculated using the following formula:

Hemolysis (%) = (A_414_ of peptide − A_414_ of PBS)/(A_414_ of Triton − A_414_ of PBS) × 100

RBCs suspended in PBS and 1% Triton X-100 represented zero hemolysis and 100% hemolysis, respectively.

### 4.7. Cytotoxicity Assay

The MTT assay was conducted to measure the cytotoxicity of the peptides towards HaCaT cells. HaCaT cells were cultured in DMEM supplemented with 1% penicillin and 10% FBS at 37 °C with CO_2_. Briefly, a total of 2 × 10^4^ cells/well were seeded into a 96-well plate, which was incubated overnight. Peptides serially diluted with DMEM at 0–64 µM were added to each well and reacted for 23 h. Next, 10 µL of 5 mg/mL MTT was added to each well, followed by incubation for 1 h. The supernatants were removed and dissolved by adding DMSO. Absorbance was measured at 570 nm [49]. The control was DMEM media without peptide. Cytotoxicity was calculated using the following formula:

Cytotoxicity (%) = 100 − [(A_570_ of peptide treated cells/A_570_ of control) × 100]

### 4.8. Biofilm Inhibition Assay

To investigate the inhibitory effect of peptide on biofilm formation, *A. baumannii* strains were cultured in MHB [50]. Bacteria were diluted to 5 × 10^5^ CFU/mL in MHB supplemented with 0.2% glucose, and then 90 µL of the bacterial suspension was mixed with 10 µL of the peptide in a 96-well plate for 24 h. The supernatant was then carefully removed, and the formed biofilm was fixed with 100% methanol for 10 min. After removing the methanol, the biofilms were stained with 0.1% crystal violet for 30 min. The plates were then washed with distilled water three times. Finally, the biofilms were completely dissolved in 95% ethanol and absorbance was measured at 595 nm using a Versa-Max microplate ELISA reader (Molecular Devices, Sunnyvale, CA, USA).

### 4.9. Biofilm Eradication Assay

To measure the removal effect of the formed biofilms, 100 µL aliquots of *A. baumannii* 907233 (5 × 10^5^ CFU/mL) were incubated in MHB with 0.2% glucose and incubated for 24 h. The culture medium was removed, and the wells were carefully washed with PBS to remove planktonic bacteria. Peptide or antibiotics were added at up to 256 µM in MHB supplemented with 0.2% glucose for 24 h. The biofilms were stained with crystal violet for 30 min, washed three times with PBS, and dissolved in 95% ethanol.

### 4.10. Visualization of Biofilms

To visualize the inhibition and elimination effect of the peptide on the biofilm, SYTO9 dye was used [51]. The formed *A. baumannii* 907233 biofilm was fixed with 100% methanol and stained with SYTO9 dye for 30 min in the dark. Images were obtained using an EVOS FL Auto 2 fluorescence microscope (Invitrogen, Carlsbad, CA, USA).

### 4.11. Salts Sensitivity Assay

*Acinetobacter baumannii* KCTC 2508 and *A. baumannii* 907233 strains were diluted to 2 × 10^5^ CFU/mL in MHB media. The peptide was serially diluted from 0 to 32 µM in the presence of physiological salts. The final concentrations of physiological salts were as follows: 50, 100, 150 mM NaCl, 0.5, 1, 2 mM MgCl_2_, and 2, 4, 8 µM FeCl_3_. After these treatments, the procedures were same as used for the MIC assay described above.

### 4.12. Mechanism of Action Analysis

Outer membrane permeabilization assay. Permeation of the bacterial outer membrane by the peptide was measured by conducting a 1-*N*-phenylnaphthylamine (NPN) uptake assay [52]. *Acinetobacter baumannii* KCTC 2508 and *A. baumannii* 907233 strains were cultured in MHB, washed, and suspended to 0.25 at OD 600 nm in 5 mM HEPES buffer. Each strain was mixed with NPN to a final concentration of 10 µM. Next, 50 µL of peptide (1×, 2×, or 4× MIC) was added to the mixture in a 96-well plate. The relative fluorescence intensity was measured over time using a Spectramax M3 spectrophotometer (Molecular Devices) at 420 nm.

Cytoplasmic membrane depolarization assay. The membrane potential-sensitive dye DiSC_3_-5 was used to measure cytoplasmic membrane depolarization [53]. *Acinetobacter baumannii* KCTC 2508 and *A. baumannii* 907233 strains were cultured in MHB and washed three times with 5 mM HEPES (pH 7.3) containing 20 mM glucose. The bacteria were resuspended to 0.05 at OD 600 in buffer (5 mM HEPES, 20 mM glucose, and 100 mM KCl) and incubated with 1 µM DiSC_3_-5. The fluorescence was stabilized for 1 h and the peptides were added to the mixture. The fluorescence was measured using a Spectramax M3 spectrophotometer with excitation at 622 nm and emission at 670 nm.

## 5. Conclusions

In this study, we confirmed that magainin 2 has an α-helix structure and showed strong antibacterial activity against *A. baumannii* including multidrug-resistant strains. Magainin 2 is not toxic towards mammalian cells and maintained its stability and excellent antibacterial activity in a biologically relevant salt environment. Magainin 2 is also effective for treating infections by inhibiting or eliminating biofilm formation. The mechanism of action of standard and drug-resistant strains is that magainin 2 acts on both the outer and inner membranes. Taken together, magainin 2 may be useful as a new antibacterial and antibiofilm agent.

## Figures and Tables

**Table 1:** Amino acid sequence and properties of magainin 2.

Name	Sequence	Molecular Mass (Da)	H	µH	Net Charge
Magainin 2	GIGKFLHSAKKFGKAFVGEIMNS-NH_2_	2465.9	0.373	0.475	+3

H: Hydrophobicity; µH: Hydrophobic moment.

**Table 2:** Mean residual ellipticity at 222 nm ([θ]_222_) and percent α-helical contents of magainin 2 in various solutions.

	Buffer	TFE				SDS			
	10 mM SP	20%	30%	40%	50%	5 mM	10 mM	20 mM	30 mM
[θ]_222_	−700.8	−5128.3	−13,207.4	−11,989	−19,749.5	−10,184.8	−11,782	−13,227.2	−13,974.2
% α-helix	RC	6.4	30.9	27.2	50.8	21.8	26.6	31.0	33.3

10 mM SP: 10 mM Sodium Phosphate buffer, pH 7.2.

**Table 3:** Minimum inhibitory concentration (MIC) of peptides and conventional agents against *Acinetobacter baumannii* strains.

Microorganisms	Minimum Inhibitory Concentration (µM)				
	Magainin 2	Buforin 2	Melittin	Ciprofloxacin	Gentamicin
*A. baumannii* KCTC 2508	4	8	2	2	4
*A. baumannii* 244752	2	8	2	256	>256
*A. baumannii* 409081	2	8	1	>256	>256
*A. baumannii* 719705	2	8	2	128	>256
*A. baumannii* 892199	2	16	2	256	>256
*A. baumannii* 907233	2	16	2	128	>256

**Table 4:** MIC values of peptides in the presence of physiological salts.

Salt	Concentration	*A. baumannii* KCTC 2508		*A. baumannii* 907233	
		Magainin 2	Buforin 2	Magainin 2	Buforin 2
NaCl	50 mM	4	16	2	32
	100 mM	4	32	4	>32
	150 mM	4	>32	4	>32
MgCl_2_	0.5 mM	4	16	4	>32
	1 mM	8	32	8	>32
	2 mM	8	>32	8	>32
FeCl_3_	2 µM	4	4	2	16
	4 µM	4	4	2	16
	8 µM	4	4	2	16
